# The Archives of Pedriatics

**Published:** 1895-11

**Authors:** 


### The Archives of Pediatrics

Will commence its thirteenth year with the January number, under the business management of E. B. Treat, Publisher, of New York, long identified with medical publishing interests. The *Archives* has been for twelve years the only journal in the English language devoted exclusively to "Diseases of Children," and has always maintained a high standard of excellence.

The new management propose several important changes in its make-up; increasing the text fifteen per cent., and enlarging its scope in every way. This will give room for the fuller contributions and additional collaborators who have been secured for the various departments, all of which give promise of a more successful era than has been known even in the already brilliant career of the journal.

The editorial management will be in the hands of Floyd M. Crandall, M. D., Adjunct Professor of Pediatrics, New York Polyclinic, and Chairman of Section on Pediatrics, New York Academy of Medicine.

